# A Randomized Placebo-Controlled Efficacy Study of a Prime Boost Therapeutic Vaccination Strategy in HIV-1-Infected Individuals: VRI02 ANRS 149 LIGHT Phase II Trial

**DOI:** 10.1128/JVI.02165-20

**Published:** 2021-04-12

**Authors:** Y. Lévy, C. Lacabaratz, E. Lhomme, A. Wiedemann, C. Bauduin, C. Fenwick, E. Foucat, M. Surenaud, L. Guillaumat, V. Boilet, V. Rieux, O. Bouchaud, P.-M. Girard, J.-M. Molina, P. Morlat, L. Hocqueloux, L. Richert, G. Pantaleo, J. D. Lelièvre, R. Thiébaut,

**Affiliations:** a Vaccine Research Institute-VRI, Hôpital Henri Mondor, Créteil, France; b INSERM, Unité U955, Créteil, France; c Université Paris-Est, Faculté de Médecine, UMR-S 955, Créteil, France; d Assistance Publique-Hôpitaux de Paris (AP-HP), Groupe Henri-Mondor Albert-Chenevier, Service d’Immunologie clinique, Créteil, France; e University of Bordeaux, Department of Public Health, Inserm Bordeaux Population Health Research Centre, Inria SISTM, Bordeaux, France; f Pôle de Santé Publique, CHU de Bordeaux, Bordeaux, France; g University of Bordeaux, ISPED, Inserm, Population Health Research Center, Team MORPH3EUS, UMR 1219, CIC-EC 1401, Bordeaux, France; h Division of Immunology and Allergy, Department of Medicine, Centre Hospitalier Universitaire Vaudois and University of Lausanne, Lausanne, Switzerland; i Inserm-ANRS, Vaccine Research Office, Paris, France; j Hôpital Avicenne, AP-HP, Université Paris 13, Bobigny, France; k Hôpital Saint Antoine, AP-HP, INSERM UMR S_1136, Paris, France; l Hôpital Saint-Louis, AP-HP, Université Paris Diderot INSERM U941, Paris, France; m CHU de Bordeaux, Bordeaux, France; n CHR d’Orléans, La Source, France; Emory University

**Keywords:** HIV, antiretroviral therapy interruption, therapeutic vaccine, clinical trials, human immunodeficiency virus

## Abstract

In this placebo-controlled phase II randomized clinical trial, 103 human immunodeficiency virus type 1 (HIV-1)-infected patients under cART (combined antiretroviral treatment) were randomized 2:1 to receive either 3 doses of DNA GTU-MultiHIV B (coding for Rev, Nef, Tat, Gag, and gp160) at week 0 (W0), W4, and W12, followed by 2 doses of LIPO-5 vaccine containing long peptides from Gag, Pol, and Nef at W20 and W24, or placebo. Analytical treatment interruption (ATI) was performed between W36 to W48. At W28, vaccinees experienced an increase in functional CD4^+^ T-cell responses (*P* < 0.001 for each cytokine compared to W0) measured, predominantly against Gag and Pol/Env, and an increase in HIV-specific CD8^+^ T cells producing interleukin 2 (IL-2) and tumor necrosis factor alpha (TNF-α) (*P* = 0.001 and 0.013, respectively), predominantly against Pol/Env and Nef. However, analysis of T-cell subsets by mass cytometry in a subpopulation showed an increase in the W28/W0 ratio for memory CD8^+^ T cells coexpressing exhaustion and senescence markers such as PD-1/TIGIT (*P* = 0.004) and CD27/CD57 (*P* = 0.044) in vaccinees compared to the placebo group. During ATI, all patients experienced viral rebound, with the maximum observed HIV RNA level at W42 (median, 4.63 log_10_ copies [cp]/ml; interquartile range [IQR], 4.00 to 5.09), without any difference between arms. No patient resumed cART for CD4 cell count drop. Globally, the vaccine strategy was safe. However, a secondary HIV transmission during ATI was observed. These data show that the prime-boost combination of DNA and LIPO-5 vaccines elicited broad and polyfunctional T cells. The contrast between the quality of immune responses and the lack of potent viral control underscores the need for combined immunomodulatory strategies. (This study has been registered at ClinicalTrials.gov under registration no. NCT01492985.)

**IMPORTANCE** In this placebo-controlled phase II randomized clinical trial, we evaluated the safety and immunogenicity of a therapeutic prime-boost vaccine strategy using a recombinant DNA vaccine (GTU-MultiHIV B clade) followed by a boost vaccination with a lipopeptide vaccine (HIV-LIPO-5) in HIV-infected patients on combined antiretroviral therapy. We show here that this prime-boost strategy is well tolerated, consistently with previous studies in HIV-1-infected individuals and healthy volunteers who received each vaccine component individually. Compared to the placebo group, vaccinees elicited strong and polyfunctional HIV-specific CD4^+^ and CD8^+^ T-cell responses. However, these immune responses presented some qualitative defects and were not able to control viremia following antiretroviral treatment interruption, as no difference in HIV viral rebound was observed in the vaccine and placebo groups. Several lessons were learned from these results, pointing out the urgent need to combine vaccine strategies with other immune-based interventions.

## Abstract (precis)

In this placebo-controlled phase II randomized clinical trial, we evaluated the safety and immunogenicity of a therapeutic prime-boost vaccine strategy using a recombinant DNA vaccine (GTU-MultiHIV B clade) followed by a boost vaccination with a lipopeptide vaccine (HIV-LIPO-5) in HIV-infected patients on combined antiretroviral therapy. We show here that this prime-boost strategy is well tolerated, consistently with previous studies in HIV-1-infected individuals and healthy volunteers who received each vaccine component individually.

## INTRODUCTION

Despite the beneficial effects of combined antiretroviral therapy (cART) on human immunodeficiency virus (HIV) morbidity and mortality, these drugs do not eradicate the latent HIV reservoir, resulting in a constant rebound in viremia after stopping cART (1). Several strategies are under development to clear latently infected cells, which contain integrated HIV DNA and are capable of surviving indefinitely in patients despite long-term cART. The concept behind these strategies, which needs to be proven, is that activation of these cells, for instance, by using latency-reversing agents, may lead to HIV reactivation, expression of HIV proteins, and elimination of these cells by the immune system (2). Until now, clinical outcomes using this strategy have been disappointing. One possible obstacle is that the killing of cells harboring HIV requires robust and efficient T-cell responses, making therapeutic vaccination central in strategies aiming at reducing the latent HIV reservoir and achieving a functional cure (3).

In the last 25 years, several vaccine strategies to restore and improve HIV-specific functional immune responses have been developed, with various results in terms of immunogenicity or HIV control when experimental designs comprised a period of ART interruption. In some trials, a partial effect on viral rebound was observed (4). Although promising, firm conclusions on the efficacy of these strategies are difficult to draw when they are based on noncontrolled studies (4).

In the present study, we sought to address some of these issues by designing a randomized, placebo-controlled therapeutic vaccination trial combining two different HIV vaccines, GTU-MultiHIV B clade and long HIV lipopeptide sequences (HIV LIPO-5), in a prime-boost regimen. These two vaccines share homologous HIV sequences and strong cytotoxic T lymphocyte (CTL) epitopes. The GTU-MultiHIV B vaccine encodes a MultiHIV antigen (Rev, Nef, Tat, Gag p17/p24 proteins, and an epitope stretch of previously identified CTL epitope-rich regions encoded by *pol* and *env* of a subtype B HIV-1 isolate, Han-2), and the lipopeptides are composed of 5 synthetic peptides (Nef 66 to 97, Nef 116 to 145, Gag 17 to 35, Gag 253 to 284, and Pol 325 to 355, also from a clade B strain) to which lipid chains are covalently bound. GTU-MultiHIV B has been evaluated in untreated HIV patients, where it led to an HIV-specific sustained CD4^+^ and CD8^+^ T-cell response, as well as a significant decline in plasma HIV viral load (5). More recent results combining transcutaneous (t.c.) and intramuscular (i.m.) injection of GTU-MultiHIV B showed a lack of improvement of immune responses in treated HIV patients, leading to the conclusion of the need of a combinatory approach (6). LIPO-5 has been evaluated in healthy adults, where it led to sustained HIV-specific CD4^+^ and CD8^+^ T-cell responses (7, 8).

Correlates of protection/control/cure are supposed to be different from HIV prophylactic interventions, and T-cell responses in particular are supposed to play a key role in the clearance of infected cells. Successful approaches in simian immunodeficiency virus (SIV), such as the cytomegalovirus (CMV)-based vaccine, have given a new basis to the key role of CD8^+^ T-cell response (9). There is a consensus in the field to propose antiretroviral treatment interruption (ATI) for evaluation of the virological efficiency of an immune intervention on the HIV reservoir, since the ultimate objective of any intervention in treated HIV patients is to maintain low viral replication after cART withdrawal (10). The efficacy endpoints of our study included T-cell immunogenicity and viral kinetics following a 12-week period of ATI.

## RESULTS

### Study participants.

**FIG 1:**
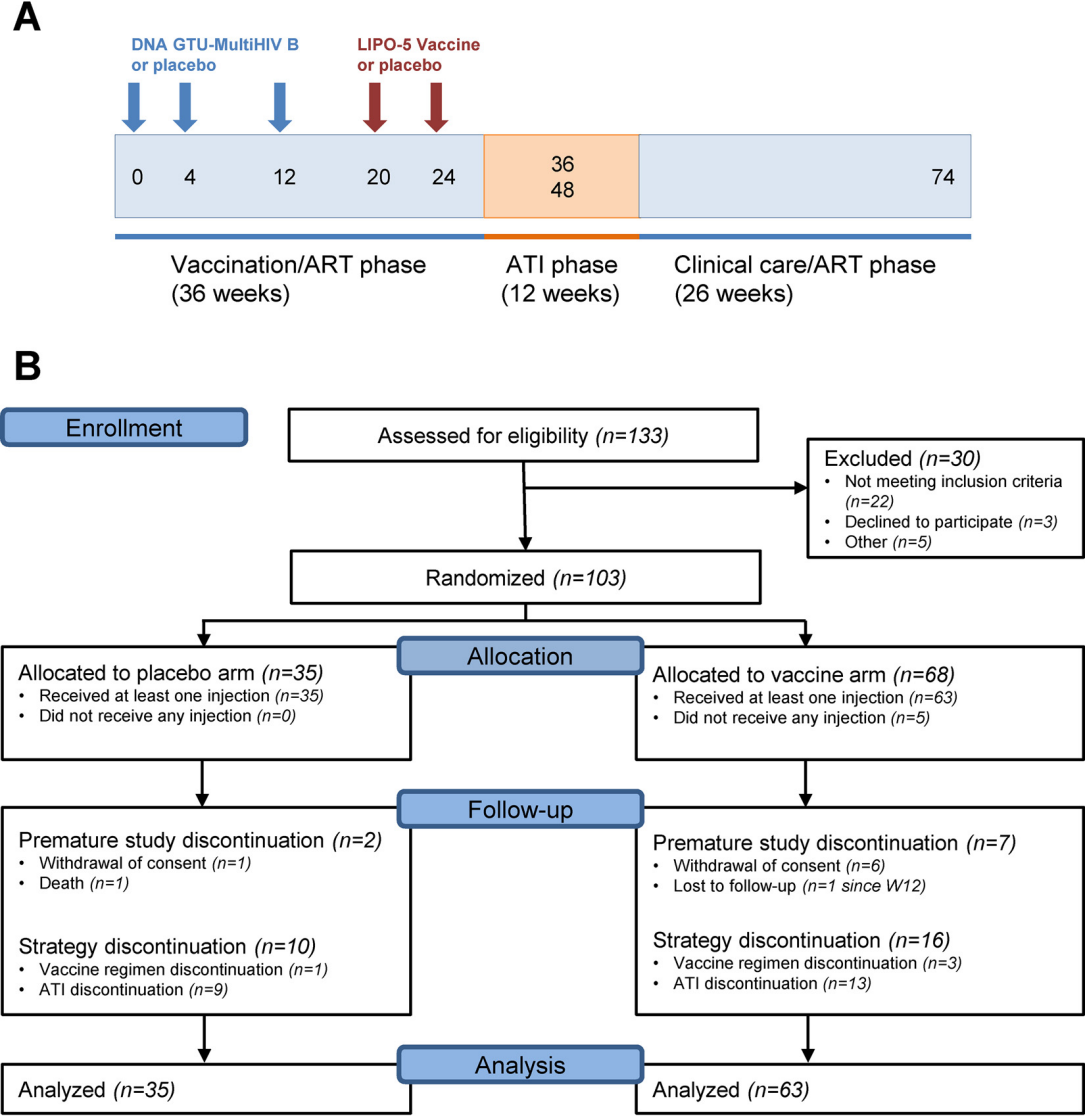
Trial design. (A) Schematics of study design. Blue arrows indicate time of DNA GTU-MultiHIV B or placebo administrations. Red arrows indicate time of HIV LIPO-5 or placebo administrations. ART, antiretroviral therapy; ATI, analytical treatment interruption; (B) Consolidated Standards of Reporting Trials (CONSORT) flow diagram for the trial. CONSORT diagram delineates the study enrollment of 103 participants who underwent randomization to the placebo or vaccine groups.

In total, 133 HIV-infected individuals were screened, and 103 were enrolled and randomized in 18 centers in France between September 2013 and May 2015 (Fig. 1B). Five participants withdrew consent before receiving any intervention, and 98 received at least one injection of placebo (*n* = 35) or vaccine strategy (*n* = 63) and were included in the modified intention to treat (mITT) analysis of the study. Baseline characteristics of participants are reported in Table 1. The two study arms were balanced at baseline. Nine participants (2 and 7 in the placebo and vaccine groups, respectively) withdrew from further follow-up after week 0, the majority due to consent withdrawal.

**TABLE 1:** Baseline characteristics of study participants

Participant characteristic	Placebo (*n* = 35)	Vaccine (*n* = 63)	Total (*n* = 98)
Age (yrs)^*a*^	44 (38; 49)	46 (36; 51)	45 (38; 51)
Male, *n* (%)	30 (86)	56 (89)	86 (88)
Time since first positive serology (in yrs)^*a*^	7 (5; 13)	8 (4; 14)	7 (4; 14)
Nadir CD4^+^ count (per mm^3^)^*a*^	390 (335; 502)	389 (332; 480)	390 (334; 480)
CD4^+^ count at baseline (per mm^3^)^*a*^	844 (684; 1,060)	840 (744; 1,018)	842 (733; 1,045)
RNA zenith (log_10_ cp/ml)^*a*^	5.1 (4.8; 5.6)	5.0 (4.4; 5.4)	5.0 (4.5; 5.4)
RNA at baseline (log_10_ cp/ml)^*a*^	1.6 (1.6; 1.6)	1.6 (1.6; 1.6)	1.6 (1.6; 1.6)

a Median (Q1; Q3).

### Global overview of vaccine immunogenicity.

**FIG 2:**
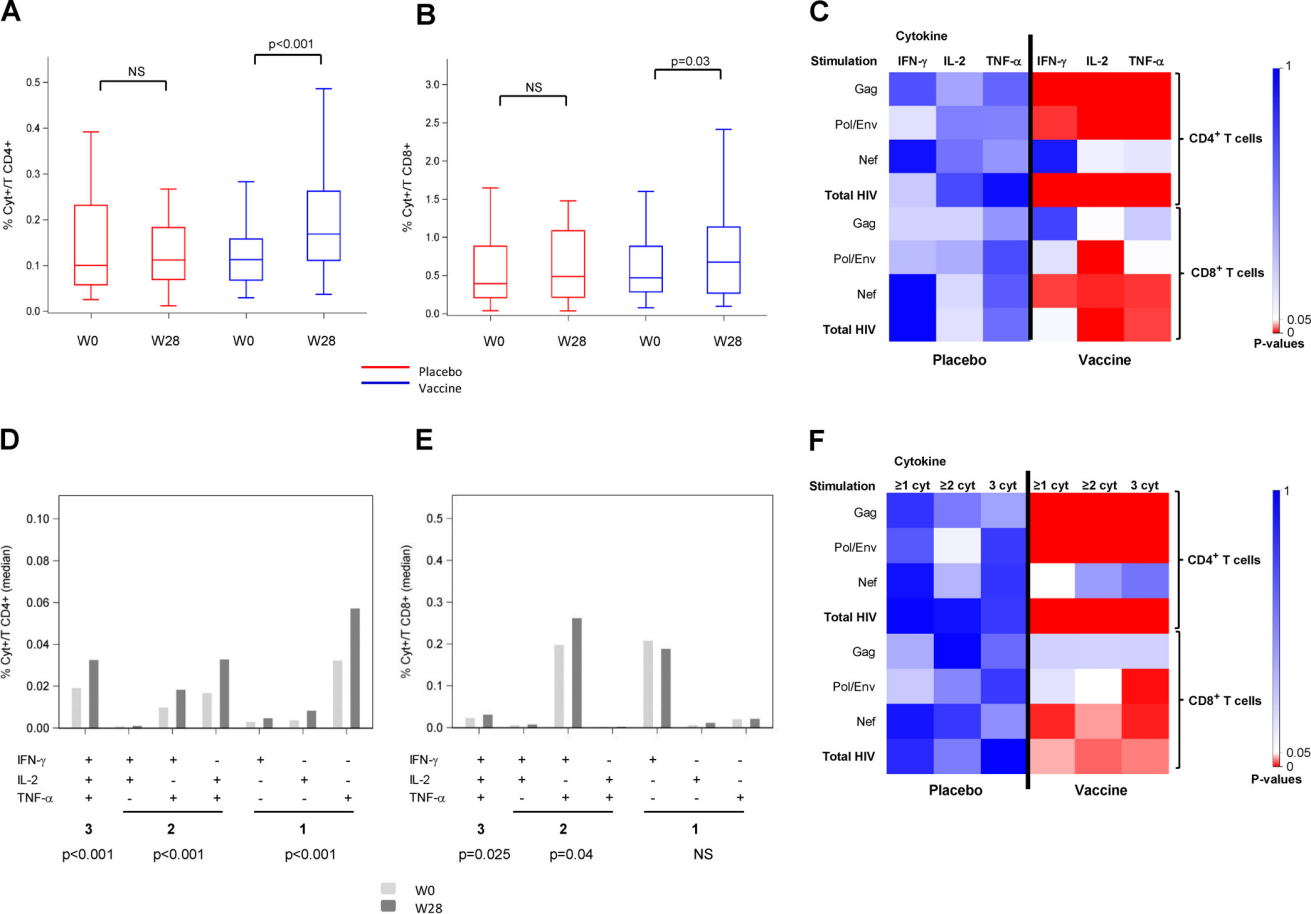
Functional profile of CD4^+^ and CD8^+^ T-cell responses. Production of interleukin 2 (IL-2), gamma interferon (IFN-γ), and tumor necrosis factor alpha (TNF-α) as measured by intracellular cytokine staining (ICS) using multiparametric flow cytometry after cell stimulation before (W0) and after vaccination (W28) in placebo (red) and therapeutic vaccine (blue) groups. (A) HIV-specific CD3^+^ CD4^+^ T-cell frequency; (B) HIV-specific CD3^+^ CD8^+^ T-cell frequency; (C) heatmap of *P* values between W28 and W0 of CD3^+^ CD4^+^ and CD3^+^ CD8^+^ marginal responses against Gag, Pol/Env, Nef, and the sum of HIV peptides (total HIV); (D) frequency of HIV-specific CD3^+^ CD4^+^ T cells producing 1, 2, or 3 cytokines in the vaccine group at W0 (light gray) and W28 (dark gray); (E) frequency of HIV-specific CD3^+^ CD8^+^ T cells producing 1, 2, or 3 cytokines in the vaccine group at W0 (light gray) and W28 (dark gray); (F) heatmap of *P* values between W28 and W0 of CD3^+^ CD4^+^ and CD3^+^ CD8^+^ polyfunctionality responses against Gag, Pol/Env, Nef, and total HIV peptides.

Evaluation of vaccine-elicited T-cell responses measured by flow cytometry-based intracellular cytokine staining (ICS) for antigen-specific gamma interferon (IFN-γ) and/or interleukin 2 (IL-2) and/or tumor necrosis factor alpha (TNF-α) was performed at baseline and week 28 on 92 participants who received the complete schedule of vaccination/placebo until week 28 in a per-protocol analysis. Total CD4^+^ T cell responses to several HIV peptide pools showed no difference between groups at entry. We found a significant increase of CD4^+^ T cells producing cytokines against HIV peptide pools in the vaccine group at week 28 compared to baseline (*P* < 0.001), while no difference was observed in the placebo group (Fig. 2A). Detailed analysis for each cytokine and each HIV peptide pool showed a significant increase in IFN-γ, IL-2, and TNF-α production after Gag and Pol/Env stimulation, but not that by Nef, in the vaccine group (Fig. 2C), as well as increases of CD4^+^ T cells producing 1, 2, or 3 cytokines at W28 (*P* < 0.001 for all comparisons to baseline) (Fig. 2D), specially against Gag and Pol/Env pools (*P* < 0.001 for each comparison) (Fig. 2F). While CD8^+^ T cells producing cytokines against HIV peptides did not change in the placebo group, we found an increase of total cytokines in the vaccine group at week 28 compared to baseline (Fig. 2B), and detailed analysis showed that these responses were directed against Pol/Env and Nef peptides, but not against Gag (Fig. 2C). The frequency of polyfunctional CD8^+^ T cells (producing at least 2 cytokines), but not that of monofunctional CD3^+^ CD8^+^ T cells (producing only 1 cytokine), increased significantly in the vaccine group compared to W0 (*P* = 0.04 and 0.025 for production of 2 and 3 cytokines, respectively) (Fig. 2E), especially after Pol/Env or Nef stimulation (Fig. 2F).

**FIG 3:**
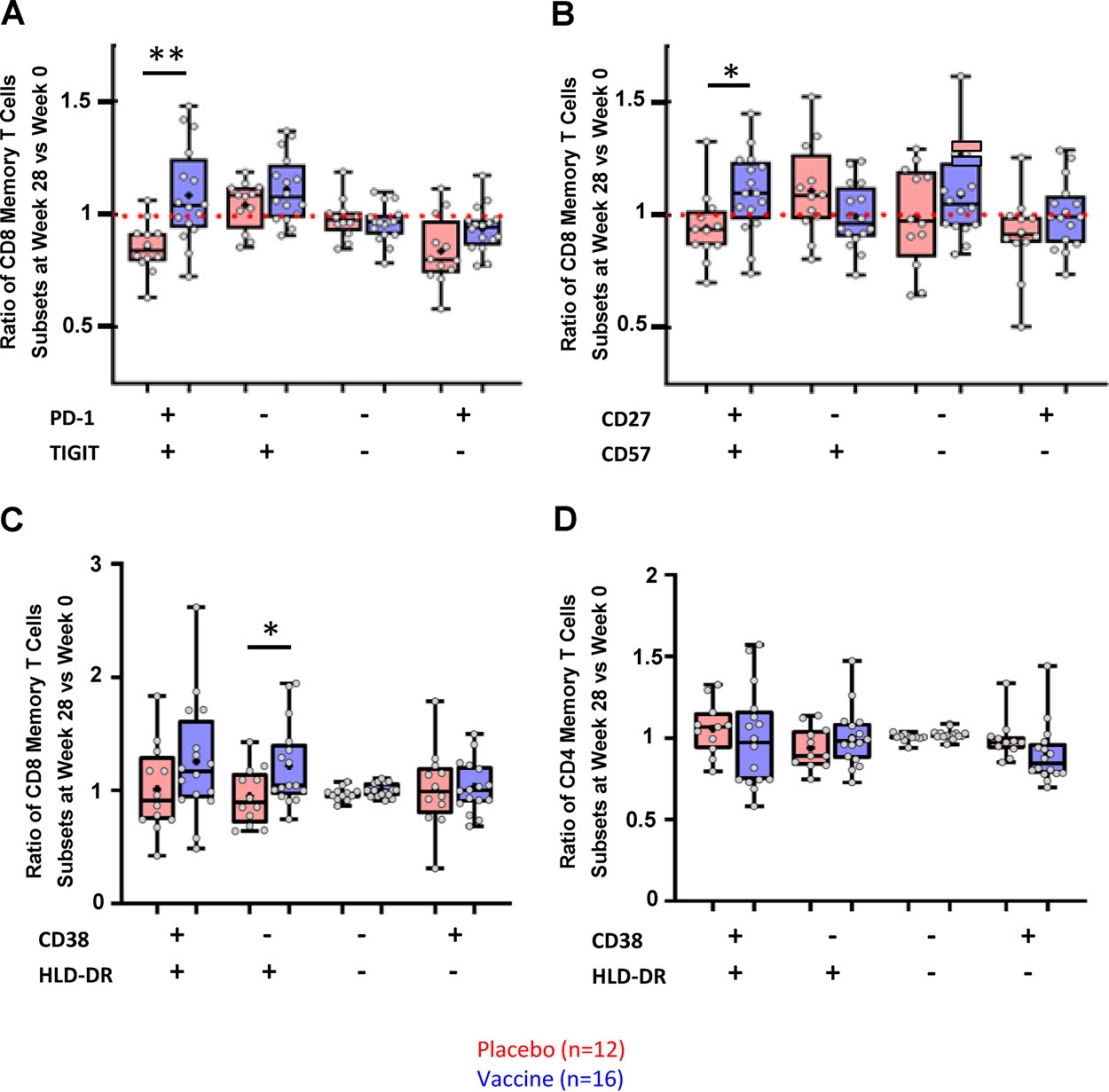
Mass cytometry (CyTOF) phenotyping. Ratio of memory CD8^+^ T cells at W28 compared to W0 for several subsets according to PD-1 and TIGIT (A), CD27 and CD57 (B), or HLA-DR and CD38 (C) in placebo (red) and therapeutic vaccine (blue) groups. Ratio of memory CD4^+^ T cells at W28 compared to W0 for HLA-DR and CD38 (D) in placebo (red) and therapeutic vaccine (blue) groups. *P* values were calculated using the Mann-Whitney test; *, *P* = 0.017; **, *P* = 0.0022.

To extend the analysis of immune cells, an ancillary analysis of T-cell phenotypic profile was performed in 28 patients (12 placebo and 16 vaccinees), with mass cytometry allowing the detection of 40 cell surface markers. Figure 3 depicts the W28/W0 ratio of gated positive populations for each marker in vaccinees compared to placebo. Significant changes of CD8^+^ memory T-cell subsets were observed in the vaccine group after vaccination, with higher frequencies of memory CD8^+^ T cells coexpressing PD-1 and TIGIT (Fig. 3A) and coexpressing CD27 and CD57 (Fig. 3B). Changes in the population of CD8^+^ T cells exhibiting markers of activation were also observed, as memory HLA DR^+^ CD38^−^ CD8^+^ T cells were increased in the vaccine group (Fig. 3C) without any modification of the memory CD4^+^ T cells (Fig. 3D). No change was observed in the different CD4^+^ T-cell subsets, including Treg or CD32a^+^ expressed on a CD4^+^ T-cell HIV reservoir (data not shown).

### Analytical treatment interruption.

**FIG 4:**
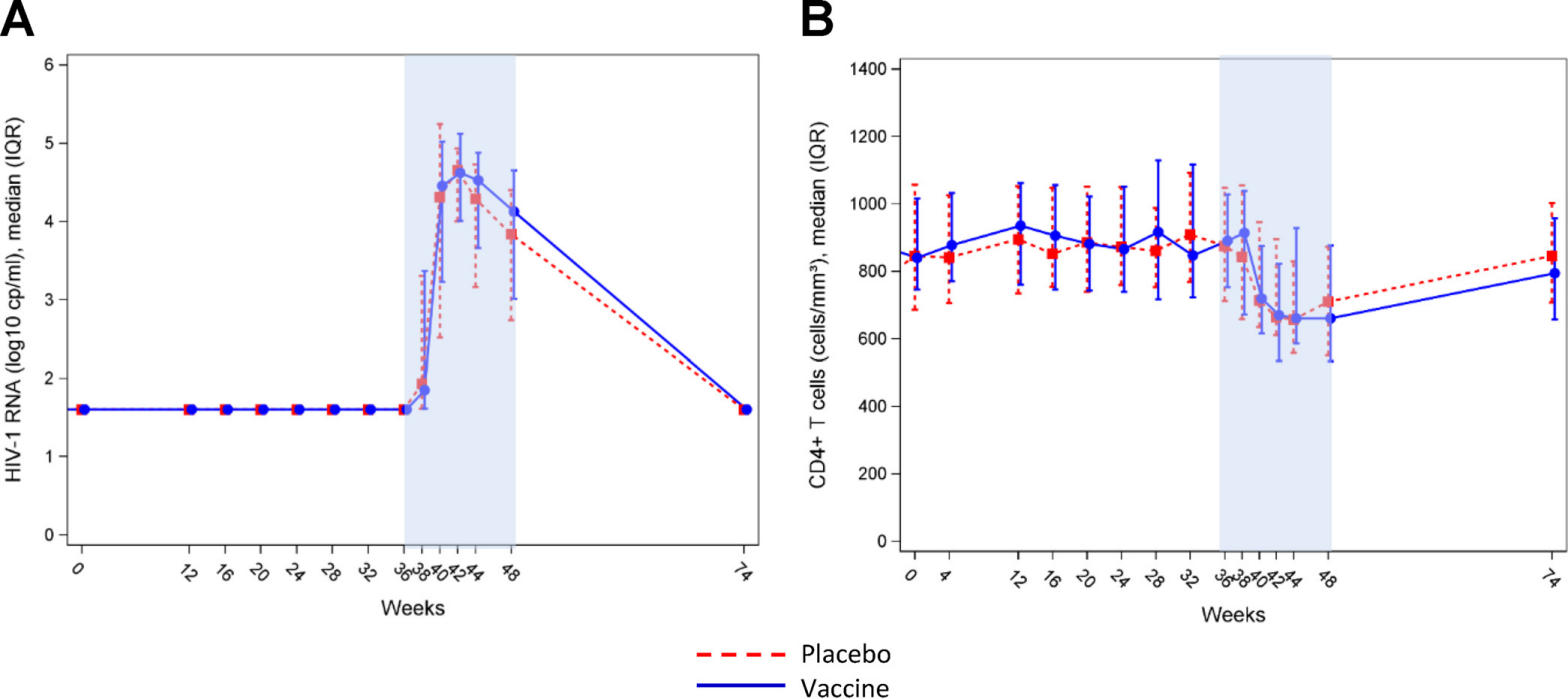
Plasma HIV viral load and CD4^+^ T-cell count changes throughout the study. (A) Levels of plasma HIV RNA in the placebo (red) and therapeutic vaccine (blue) groups before and after ATI (weeks 36 to 48); (B) CD4^+^ T-cell count changes during the vaccination phase and following ATI in the placebo (red) and therapeutic vaccine (blue) groups before and after ATI (weeks 36 to 48).

ATI was proposed in both arms to individuals with plasma HIV RNA concentrations of <50 copies (cp)/ml at week 36. A total of 89 participants (*n* = 32 [91%] and 57 [90%] in placebo and vaccine groups, respectively) started ATI according to the study protocol and were followed until week 48, the final study endpoint. In total, 65 participants (*n* = 23 [66%] and 42 [67%] in placebo and vaccine groups, respectively) resumed ART according to the study protocol at week 48. A total of 18 participants (*n* = 7 [20%] and 11 [17%] in placebo and vaccine groups, respectively) resumed ART before week 48 for participants or doctors’ decisions. Two participants from each group resumed ART after week 48, and two participants from the vaccine group did not resume ART at the end of the follow-up after week 48. Figure 4A shows longitudinal evolution of HIV plasma viral loads (VL) in the two study arms during the ATI period. The maximum level of viral load was observed at week 42 in both groups. mITT analysis did not show any significant differences between groups in terms of the maximum observed (peak) viral load; median peak VL (first quartile, Q1; third quartile, Q3) between weeks 36 and 48 were 5.26 (4.58; 7) and 5.15 (4.73; 7) in the placebo and vaccine groups, respectively (*P* = 0.9). The frequency of participants with a VL below 10,000 copies/ml at week 48, defined as virological success, was 50% and 44% in the placebo and vaccine groups, respectively. In total, 71 patients met this predefined success criterion of the strategy without any significant difference between groups, i.e., 25 and 46 in the placebo and vaccine groups, respectively (Table 2).

**TABLE 2:** Plasma HIV RNA values during ATI period

Parameter	Placebo (*n* = 35)	Vaccine (*n* = 63)	Total (*n* = 98)
Maximum VL during ATI (log_10_ cp/ml)^*a*^
Mean (SD)	5.39 (1.40)	5.42 (1.17)	5.41 (1.25)
Median (IQR)	5.26 (4.58–7.00)	5.15 (4.73–7.00)	5.16 (4.70–7.00)
Range	1.60–7.00	1.60–7.00	1.60–7.00
ATI experience between W36 and W48, *n* (%)	25 (71)	46 (73)	71 (72)
Time of maximum VL during ATI in participants having experienced ATI, *n* (%)
W38	2 (8)	3 (7)	5 (7)
W40	11 (44)	13 (28)	24 (34)
W42	8 (32)	15 (33)	23 (32)
W44	2 (8)	9 (20)	11 (15)
W48	2 (8)	6 (13)	8 (11)
Maximum VL during ATI (log_10_ cp/ml) in participants having experienced ATI	6.12	5.95	6.12
Participants with VL below 10,000 cp/ml at W48^*b*^ (*n* [%])
No	17 (50)	34 (56)	51 (54)
Yes	17 (50)	27 (44)	44 (46)

a *P* value for comparison of placebo vs. vaccine = 0.878.

b Four participants did not resume ART at W36 and were considered in virological failure at W48.

The kinetics of the peak of VL look slightly different between groups that experienced ATI. At week 40, the maximum peak of VL was observed in 44% and 28% of participants in the placebo and vaccine groups, respectively (*P* = 0.27) and in 8% and 20% of participants in the placebo and vaccine groups, respectively (Table 2). At the end of the ATI phase (week 48), two participants from the vaccine group did not resume ART because of plasma VL below 50 copies/ml. These participants had an initial VL rebound at weeks 42 and 44 and then exhibited a spontaneous suppression of viremia, which remained undetectable without ART at the end of the study. Among the 91 participants restarting cART, 75 (31 and 44 in the placebo and vaccine groups, respectively) participants showed suppressed viremia (<50 copies/ml) at W74.

Figure 4B shows patterns of CD4^+^ T-cell changes in participants during the ATI period. The evolution was similar in both groups of participants, with a nadir (median [interquartile range (IQR)]) at week 44 of 657 cells/mm^3^ (556-832) and 661 (584-930) in the placebo and vaccine groups, respectively. CD4^+^ T-cell counts remained similar in both groups at the end of the study and after resuming ART at week 48.

### Relationship between polyfunctionality of HIV-specific T-cell responses and viral parameters following ATI.

A principal-component analysis (PCA) was conducted to illustrate the interrelationships between vaccine-induced T-cell responses measured by ICS before ATI (polyf CD4 and polyf CD8) and viral parameters during ATI.

**FIG 5:**
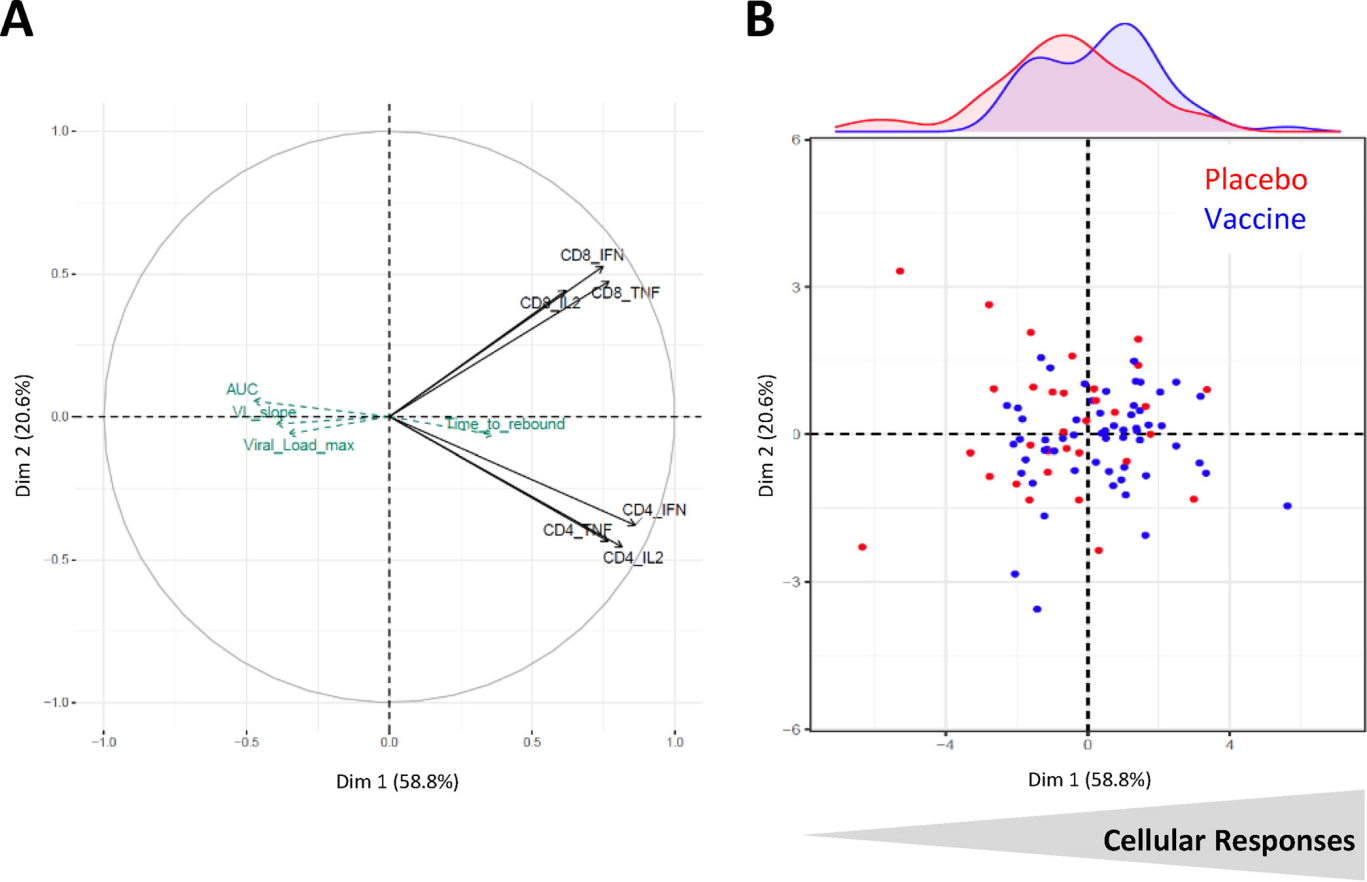
Integrative analysis of immune response to vaccine. Principal-component analysis of ICS responses at W28. Log-transformed marginal CD4^+^ and CD8^+^ T-cell responses at W28 were included as active variables; virological markers during ATI (highest viral load, viral load slope, viral load area under the curve [AUC], and time to rebound) were included as supplementary variables. (A) Projection of variables; (B) projection of individuals represented into placebo (red) and therapeutic vaccine (blue) groups.

Figure 5A is a projection of variables on the first two axes. The first principal component (*x* axis) represented 59% of the variability, while the second principal component represented 21%. All immunological variables were on the right side of the figure, illustrating their trend to be positively correlated. The *y* axis allowed differentiation of the CD8^+^ T-cell responses (top) and the CD4^+^ T-cell responses (bottom). Results showed that maximal viral load, viral load slope, and viral load area under the curve (AUC) were projected at the opposite direction of the immune markers, indicating a trend toward negative correlations between the magnitude of viral load after ATI and T-cell responses before ATI. In Fig. 5B, the representation of the patients on the PCA illustrates the poor immunological status of some patients (left) and the CD4^+^ and CD8^+^ T cell-oriented response of the others (middle right). Vaccinated participants seemed to be slightly more numerous on the right part of the plan (as shown by the blue distribution curve on the top), corresponding to good responders, while those on the left part exhibited poorer immunological responses (being vaccinated or not) with a higher maximum viral load.

### Safety.

In total, 98 individuals received at least one injection, and 93 received all injections. The majority of participants (96%) experienced at least one adverse event (AE) that was transient (median duration, 15 days; IQR 3-62). As shown in Table 3, most of the AEs were grade 1 or 2, and there were no marked differences between arms. Among 15 serious adverse events (SAEs) (Table 4), one was possibly related to the GTU-Multi-HIV B vaccine (arthritis) and one to the research, i.e., secondary HIV transmission during the ATI period, confirmed by phylogenetic analysis of the HIV in the placebo arm (11). Primary infection-like symptoms, usually mild, were observed in 23% of the individuals after ATI. There was no resumption of ART due to CD4 cell count drop during ATI.

**TABLE 3:** Adverse events after W0

Parameter	Placebo (*n* = 35)	Vaccine (*n* = 63)	Total (*n* = 98)
No. (%) of participants presenting			
At least one AE	35 (100)	59 (94)	94 (96)
At least one biological AE	5 (14)	9 (14)	14 (14)
At least one clinical AE	35 (100)	59 (94)	94 (96)
AE by maximal grade, *n* (%)	220	405	625
Grade 1: mild	84 (38)	217 (54)	301 (48)
Grade 2: moderate	123 (56)	170 (42)	293 (47)
Grade 3: severe	12 (5)	18 (4)	30 (5)
Grade 4: life threatening	1 (0)	0	1 (0)
SAE among all AEs, *n* (%)			
No	213 (97)	397 (98)	610 (98)
Yes	7 (3)	8 (2)	15 (2)
Median duration of AE (in days) (Q1; Q3)	24 (5; 90)	12 (3; 49)	15 (3; 62)
Participants presenting at least one AE related to vaccine, *n* (%)	12 (34)	34 (54)	46 (47)
AE related to vaccine by maximal grade, *n* (%)	36	105	141
Grade 1: mild	19 (53)	85 (81)	104 (74)
Grade 2: moderate	17 (47)	19 (18)	36 (26)
Grade 3: severe	0	1 (1)	1 (1)
Grade 4: life threatening	0	0	0
SAE among AE related to vaccine, *n* (%)			
No	36 (100)	104 (99)	140 (99)
Yes	0	1 (1)	1 (1)

**TABLE 4:** Description of severe adverse events (SAE) after W0 by system organ class and preferred term

System organ class (SOC)	Preferred term (PT)	*n* (%)		
		Placebo (*n* = 7)	Vaccine (*n* = 8)	Total (*n* = 15)
Infections and infestations	Peritonitis	1 (14.3)	1 (6.7)
	Bacterial rectitis	1 (12.5)	1 (6.7)
	Transmitting the HIV infection	1 (14.3)	1 (6.7)
Injury, poisoning, and procedural complications	Accident on the public highway	1 (12.5)	1 (6.7)
	Artery stenosis	1 (12.5)	1 (6.7)
	Toxicity of various agents	1 (12.5)	1 (6.7)
Musculoskeletal and connective tissue disorders	Arthralgia	1 (14.3)	1 (6.7)
	Rheumatoid arthritis	1 (12.5)	1 (6.7)
Heart disorders	Congestive cardiomyopathy	1 (14.3)	1 (6.7)
Reproductive system and breast disorders	Benign prostatic hypertrophy	1 (14.3)	1 (6.7)
Nervous system disorders	Craniocerebral injuries plus loss of consciousness	1 (12.5)	1 (6.7)
Blood and lymphatic system disorders	Iron deficiency anemia	1 (14.3)	1 (6.7)
Psychiatric disorders	Suicide	1 (14.3)	1 (6.7)
Respiratory, thoracic, and mediastinal disorders	Pulmonary disorder	1 (12.5)	1 (6.7)
Metabolism and nutrition disorders	Diabetes	1 (12.5)	1 (6.7)

## DISCUSSION

In this study, we show that a therapeutic immunization strategy combining a DNA prime followed by a boost with HIV long lipopeptides is well tolerated in chronically HIV-1-infected individuals treated with cART. These safety data are consistent with previous studies in HIV-1-infected individuals and healthy volunteers who received each vaccine component individually (8, 12, 13).

This study comprised two phases, a vaccination period followed by an ATI phase of 12 weeks, to evaluate both the immunogenicity and virologic efficacy of the vaccine strategy.

At the end of the vaccination period, the immunogenicity of the vaccine strategy was clearly demonstrated. Vaccinees exhibited significant changes in the frequency and the functionality of HIV-specific T-cell responses. However, these changes in the immune status of individuals did not translate into any differences in the kinetics and magnitude of viral rebound following ATI. Consistently, we found that the vaccine strategy did not significantly impact the levels of cellular HIV-DNA measured before ATI (14). Nevertheless, integrative analysis of virological and immunological parameters showed a trend toward an association between good vaccine responders and a lower viral load after ATI, while individuals with poorer immunological responses (being vaccinated or not) exhibited a higher maximum viral load.

These findings might have important implications in the design and evaluation of future studies testing immunological interventions aimed at sustainably control viral replication without cART.

The rationale to combine a DNA GTU prime and HIV long lipopeptides was based on previous results obtained with each individual vaccine component. Administration of DNA GTU in cART-naive individuals resulted in a modest, but significant, decrease of plasma HIV viral load (up to 0.5 log_10_ copies/ml) in a large therapeutic study performed in South African individuals (5). Previous therapeutic vaccine studies centered around HIV lipopeptides also provided encouraging results (7, 8). Combination of ALVAC/HIV lipopeptide and IL-2 preceding ATI in chronically HIV-1-infected patients resulted in a greater chance to maintain a viral load during a 24-week ATI period (HIV RNA below 10,000 copies/ml, as predefined in the present study) compared to individuals from a control arm (12, 15). In a recent nonrandomized vaccine study, we showed that vaccination with dendritic cells (DC) *ex vivo* generated and loaded with HIV lipopeptides (the Dalia trial) elicited strong CD4^+^ and CD8^+^ T-cell responses associated with a control of viral replication following ATI in chronically HIV-1-infected individuals (16). In these two previous studies, we found a correlation between vaccine-elicited responses and the magnitude of viral replication or the frequency of individuals maintaining plasma HIV viral loads below a predefined threshold following ATI (12, 15–18).

Here, the combination of these two vaccine components in a prime-boost strategy was also supported by the sharing of several HIV T-cell epitopes in common, raising the hypothesis of a stronger induction of HIV-specific T-cell responses. Indeed, immunogenicity analysis showed a significant expansion of functional T-cell responses (producing at least two cytokines) against HIV-1 Gag, Env, and Pol antigens for CD4^+^ T cells, while CD8^+^ T cells were directed against Env, Pol, and Nef, but not Gag, antigens. Despite this broad repertoire, these responses did not significantly impact HIV replication throughout the 12 weeks of ATI. The failure to show an association between vaccine immunological efficacy and the kinetics of viral rebound raises several questions about the repertoire, the functionality of these responses and the immunological context following vaccination. In-depth analysis and epitope mapping of T-cell responses elicited by the DC-based vaccine delivering HIV lipopeptides revealed an inverse correlation between the functionality of CD4^+^ T-cell responses (production of IL-2 and IL-13), the repertoire of these responses directed against HIV Gag, Nef, and Pol dominant epitopes, and the magnitude of viral rebound (17). These results are supported by several previous studies showing that robust HIV-1-specific T-cell responses are associated with a better control of infection in long-term nonprogressors (LTNP) (19). In the present study, we did not investigate the precise repertoire of CD4^+^ T-cell responses against individual HIV epitopes. Whether the lack of antiviral effect of HIV-specific CD4^+^ T-cell responses to a vaccine regimen containing HIV lipopeptides delivered through an i.m. route, compared to DC delivery, could be explained by a difference in the immune profile (cytokine pattern) or repertoire of vaccine elicited CD4^+^ T-cell responses warrants further analyses.

We also show that the vaccine regimen elicited expansion of memory CD8^+^ T-cell responses. Surprisingly, responses against Gag epitopes contained in the DNA GTU and lipopeptide sequences were not amplified. Several teams, including our group, have shown the importance of CD8^+^ T-cell responses to Gag in the control of HIV (17). Our results, from a subgroup of individuals, also show changes in the population of CD8^+^ T cells exhibiting markers of activation (increase of memory HLA-DR^+^ CD38^−^ CD8^+^ T cells in the vaccine group) and, more importantly, markers of exhaustion (TIGIT and PD-1) and senescence (CD57), which might indicate the low capacity of these cells to control viral replication. These inhibitory immune receptors have been previously shown to regulate antiviral and antitumor CD8^+^ T-cell effector function in mouse models of lymphocytic choriomeningitis virus (LCMV) and in humans with advanced melanoma (20–22). It has been shown that TIGIT and PD-1 blockade additively increased proliferation, cytokine production, and degranulation of tumor antigen-specific CD8^+^ T cells. One limitation of this observation is that we did not look at the expression of these markers on HIV-specific CD8^+^ T cells. However, as already described in cancer patients, we cannot rule out the possibility that these specific CD8^+^ T cells would exhibit a low capacity for killing HIV-infected cells (20). Regarding the design of future studies, these results underscore the need to include functional killing assays in the evaluation of the efficacy of vaccine trials (23).

One intriguing question, beyond the results of this trial, is that of why despite the capability of eliciting strong immune responses, several candidate vaccines tested showed disappointing results and failed to control HIV replication in cART-free individuals. We, and others, have already raised the hypothesis that the balance between inflammatory responses and activation of effector T cells seems crucial in this setting (24). The deleterious association of persistent inflammation signature after vaccination with the immune response to vaccine has been reported for several vaccine platforms (25, 26), including that for HIV (24). Recently, integrative analysis of a large set of arrays (T-cell responses, cytokine production, and blood transcriptomic changes) evaluating immune responses in individuals receiving a DC/HIV lipopeptide vaccine showed that inflammatory pathways related to Toll-like receptor signaling were associated with a poorer immune response to vaccination and poorer viral control after ATI (24). The similar involvement and impact of these pathways in response to other vaccines indicates a potential broad mechanism driving the immune response to vaccines. It is likely that these data underscore the need to carefully investigate, besides the profile of effector-specific T cells, the kinetics of inflammatory responses in future vaccine studies. Furthermore, these results point out the need to develop further strategies combining vaccines with adjuvants and/or immunomodulators (3).

The lack of immune correlates or robust markers predicting virologic control implies that a period of antiretroviral treatment interruption remains necessary to assess the efficacy of immune interventions in HIV-infected patients. Our study comprised a 12-week ATI period and an arbitrary threshold of plasma viral load defining the success of the strategy (i.e., frequency of individuals maintaining a plasma viral load below 10,000 copies/ml). At 1 month following cART interruption, a greater percentage of the placebo group reached maximum peak of plasma VL compared to the vaccine group (44% and 28% of participants, respectively). At the end of the ATI phase (week 48), two participants from the vaccine group maintained a suppressed viral load below 50 copies/ml and remained without cART at week 74. Interestingly, these participants had an initial VL rebound at weeks 42 and 44, which makes it unlikely that these two subjects were elite HIV controllers. However, we were unable to demonstrate the efficacy of vaccine regimen in an intention to treat analysis and according to the predefined criteria of success. This underscores the added value of the comparison to a well-controlled placebo group (4) to limit the risk of misinterpreting results. The decision to propose ATI to individuals receiving placebo should be carefully balanced by the risk of missing the demonstration of efficacy or of making erroneous conclusions on the existing efficacy of an immune intervention. Thus, the large heterogeneity of previous immunotherapeutic trials in terms of ATI duration, presence of a control group, threshold criteria for resuming cART, and timeline of virologic evaluation might hinder the capacity to identify promising strategies. For example, the use of a conservative criterion for resuming cART, such as plasma viremia above 1,000 to 2,000 copies/ml, risks missing important positive effects of immune interventions on viral control (27). It is likely that the recent consensus report on recommendations to optimize ATI strategies and to mitigate the risks for participants will help to better design future studies.

In order to minimize the risks for participants undergoing ATI, in our trial we used strict safety criteria for resuming cART before the end of the 12-week period of ATI, such as a confirmed >30% decline in CD4^+^ T-cell count, an absolute CD4^+^ T-cell count of <350 cells/mm^3^, or the development of acute retroviral syndrome. Globally, the strategy was well tolerated, and no individuals reached these safety criteria for resuming cART.

However, despite strong measures of counseling, ATI was associated with a secondary transmission to a sex partner of one participant from our study (11). This observation led our group to propose preexposure prophylaxis (PrEP) in our future HIV cure trial in France (28) and also at the European level (EHVA T02 trial; ClinicalTrials.gov registration no. NCT04120415 [29]). Although PrEP may mitigate the risk of secondary transmission, this strategy should be associated with strong counseling and additional measures of prevention because of the lack of clear data on the efficacy of PrEP against viral rebound to high levels of viremia following ATI. It would be also essential to closely monitor plasma viral load in participants during the ATI period and to adapt PrEP drugs to the resistance profile of the participant’s virus.

In conclusion, the prime-boost regimen tested in this study was designed to maximize the immune response and to evaluate its virologic efficacy in a well-controlled design trial that included a long-term ATI period. This study adds to the list of previous therapeutic vaccine trials showing that despite elicitation of a strong immune responses, no association with long-term control of viremia was demonstrated. However, several lessons were learned from these results, pointing out the urgent need to combine these vaccine strategies with other immune-based interventions.

## MATERIALS AND METHODS

### Study design and participants.

The VRI02 ANRS 149 LIGHT trial is a phase II randomized, placebo-controlled, double-blind, multicenter trial evaluating the safety and immunogenicity of a prime-boost vaccine strategy using a recombinant DNA prime vaccine (GTU-MultiHIV B clade) followed by a boost vaccination with a lipopeptide vaccine (HIV-LIPO-5) in HIV-infected patients on cART. Eligible patients were asymptomatic HIV-1-infected adults with CD4^+^ T-cell counts of >600 cells/μl and plasma HIV RNA counts of <50 copies/ml at screening and within the previous 6 months while on cART who were recruited in 18 hospitals in France. All study participants provided written informed consent before participation. The protocol was approved by the ethics committee of Ile de France 5 (Paris-Saint-Antoine) and authorized by the French regulatory authority (ANSM). The study is registered at ClinicalTrials.gov (registration no. NCT01492985) and EudraCT (registration no. 2009-018198-30).

### Randomization and masking.

Participants were randomized in a 1:2 ratio to receive either placebo or active vaccine. Randomization was done centrally 1 week before the first vaccination via electronic case report software (Ennov clinical software), on the basis of a randomization list generated by a statistician who was not masked to study conditions (CMG-EC, Inserm U1219, Bordeaux). Site staff and participants were both masked to the treatment assignment.

### Procedures.

DNA GTU MultiHIV and HIV LIPO-5 vaccines have been described elsewhere (5–7). Briefly, GTU-MultiHIV B clade, developed by FIT Biotech, encodes a MultiHIV antigen (synthetic fusion protein built up by full-length polypeptides of Rev, Nef, Tat, Gag p17, and p24 with more than 20 Th and CTL epitopes of protease, reverse transcriptase (RT), and Env gp160 regions of the HAN2 HIV-1 B clade). HIV-LIPO-5 vaccine consists of 5 HIV long peptides (Nef 66 to 97, Nef 116 to 145, Gag 17 to 35, Gag 253 to 284, and Pol 325 to 355) to which lipid tails are covalently bound. These lipopeptides, which cover HIV epitopes binding to >90% of HLA molecules, permit presentation of CD4^+^ and CD8^+^ T-cell epitopes, as well as generation of humoral immunity (17).

DNA GTU MultiHIV at a dose of 1 mg or placebo priming vaccinations were administered i.m. using a Biojector at study weeks 0, 4, and 12. HIV-LIPO-5 boosts at a dose of 2.5 mg (0.5 mg of each lipopeptide) or placebo were given at weeks 20 and 24 (Fig. 1A). For immunological analysis, peripheral blood mononuclear cell (PBMC) samples were collected at entry (W0), 4 weeks following the last DNA GTU prime (W16) and the last LIPO-5 boost (W28), W48 (final endpoint), and W74. A cART interruption between W36 and W48 was proposed to individuals who had HIV-1 RNA levels of <50 copies/ml and CD4^+^ T-cell counts of >600 cells/μl. Clinical, immunological (CD4^+^ and CD8^+^ T-cell counts), and virological (HIV viral load) follow-up was performed every 15 days for 2 months during ATI, then monthly. cART had to be resumed at W48 but could be resumed at any time according to the following criteria: (i) if the patients or their doctors wished to resume (ii) if the CD4^+^ T-cell count was <350 cells/μl at two consecutive measurements 2 weeks apart, and (iii) in the case of occurrence of an opportunistic infection or a serious non-AIDS defining event. Patients were followed until W74 for final safety evaluation after resuming cART.

### Intracellular cytokine staining assay.

Cell functionality was assessed by ICS, with Boolean gating to examine vaccine-induced HIV-1-specific CD4^+^ and CD8^+^ T-cell responses after stimulation with 3 different HIV 15-mer peptide pools (1 pool Gag, 1 pool Pol/Env, and 1 pool Nef peptides; JPT Peptide Technologies GmbH, Berlin, Germany). Staphylococcal enterotoxin B (SEB)-stimulated and unstimulated cells were used as positive and negative controls, respectively. The flow cytometry panel included a viability marker, CD3, CD4, and CD8 to determine T-cell lineage, and IFN-γ, TNF-α, and IL-2 antibodies. Data were acquired on a LSRFortessa 4-laser (488, 640, 561, and 405 nm) cytometer (BD Biosciences) and analyzed using FlowJo software version 9.9.4 (Tree Star, Inc.).

### Mass cytometry staining and analyses.

In a subpopulation of patients from both groups selected among those presenting an ICS response, a mass cytometry (CyTOF) analysis was performed at weeks 0 and 28. PBMCs were thawed, rested, and then stained using metal-conjugated antibodies according to the CyTOF manufacturer’s instructions (Fluidigm, San Francisco, CA). Cell viability staining was performed using the Cell-ID-103 Rh Intercallator at a final concentration of 1 μM, which was incubated with PBMCs for 15 min. PBMCs from an individual donor were treated in parallel and multiplexed for staining and mass cytometry analysis to limit sample variation due to sample preparation and analysis. Multiplexed week 0 and week 28 PBMCs were stained for 20 min with either anti-CD45 ^89^Y or anti-CD45 ^194^Pt isotopes, respectively, and then washed with CSM buffer (phosphate-buffered saline [PBS], 0.5% bovine serum albumin [BSA], and 0.02% sodium azide, all from Sigma) before combining the two samples. Pooled samples containing 2 × 10^6^ to 4 × 10^6^ cells were stained for 30 min using a cocktail of antibodies for cell surface markers in a total volume of 50 μl (Table 5). Cells were subsequently washed with CSM and PBS, fixed with 2.4% formaldehyde (Thermo Fisher) in PBS for 5 min, and then resuspended in DNA-intercalation solution (PBS, 1 μM Ir-Intercalator, 1% formaldehyde, and 0.3% saponin) before storage at 4°C until analysis. For CyTOF analysis, cells were washed 3 times with Milli-Q water and resuspended at 0.5 × 10^6^ cells/ml in 0.1% EQ four-element calibration beads solution (Fluidigm). Samples were normalized for the EQ bead intensities using the MATLAB normalizer software to limit interanalysis staining intensities. Data were processed and analyzed with Cytobank. Since W0 and W28 samples for a given donor were multiplexed and stained in parallel, the relative changes in marker intensities were determined using the W28/W0 ratio for the indicated gated positive populations.

**TABLE 5:** Overview of the mass cytometry panel

Marker	Isotope	Clone	Source	Vol (μl) per 50 μl
CD45	^89^Y	HI30	Fluidigm	0.40
CD8	^113^In	RPA-T8	BioLegend	0.50
CD4	^115^In	RPA-T4	BioLegend	0.40
CCR6	^141^Pr	11A9	Fluidigm	0.50
CD19	^142^Nd	HIB19	Fluidigm	0.80
ICOS	^143^Nd	C398.4A	BioLegend	0.80
CD69	^144^Nd	FN50	Fluidigm	0.50
CD31	^145^Nd	WM59	Fluidigm	0.60
IgD	^146^Nd	IA6-2	BD Biosciences	0.70
CD28	^147^Sm	L293	BD Biosciences	0.30
CD57	^148^Nd	G10F5	BioLegend	0.25
CCR4	^149^Sm	205410	Fluidigm	0.75
OX40	^150^Nd	ACT35	Fluidigm	1.20
CD103	^151^Eu	Ber-ACT8	Fluidigm	0.80
CD21	^152^Sm	BL13	Fluidigm	0.50
TIGIT	^153^Eu	MBSA43	Fluidigm	0.60
TLR2	^154^Sm	TL2.1	Fluidigm	1.00
CD27	^155^Gd	L128	Fluidigm	0.50
CD11c	^156^Gd	3.9	BioLegend	0.60
CCR7	^159^Tb	G043H7	BioLegend	0.30
CD14	^160^Gd	M5E2	Fluidigm	0.97
CD1c	^161^Dy	L161	BioLegend	0.30
CD32a-APC	^162^Dy	APC003	Fluidigm	3.5/1.0
CXCR3	^163^Dy	G025H7	Fluidigm	0.60
CD45RO	^165^Ho	UCHL1	Fluidigm	0.36
CD38	^167^Er	HIT2	Fluidigm	0.30
CD40L	^168^Er	24-31	Fluidigm	1.20
CD45RA	^169^Tm	HI100	Fluidigm	0.80
CD3	^170^Er	UCHT1	Fluidigm	0.40
LAG3	^172^Yb	BMS	Bio-Techne	1.60
HLA-DR	^173^Yb	L243	Fluidigm	0.30
PD1	^174^Yb	EH12.2H7	Fluidigm	0.50
CXCR4	^175^Lu	12G5	Fluidigm	0.35
CD127	^176^Yb	A019D5	Fluidigm	0.70
CD45	^194^Pt	HI30	BioLegend	0.50
CD16	^209^Bi	3G8	Fluidigm	0.50

### Study endpoints.

The primary endpoint was the maximum observed plasma HIV-1 RNA load (in log_10_ copies/ml) during the ATI period between W36 and W48. Participants not having interrupted cART at W36, or having resumed their treatment before W48, were imputed with the maximum plasma HIV-1 RNA load observed among all the participants during the ATI. A delay in reaching the maximum plasma viral load was also described in participants having experienced ATI between W36 and W48.

Clinical and virological secondary endpoints were as follows: the frequency of clinical and biological adverse events occurring during the trial; CD4^+^ T-cell counts at W40, W44, W48, and W74; HIV-1 RNA loads at W40, W44, W48, and W74; the virological success, assessed as the percentage of participants with plasma HIV-1 RNA load below 10,000 copies/ml at W48 and considering virological failure for participants not having interrupted cART at W36; the proportion of participants who reinitiated ART after W36; and proportion of participants with CD4^+^ T-cell counts of <350/mm^3^.

Secondary immunological end points were ICS based on the Boolean and the marginal percentages of cells producing IFN-γ, IL-2, and TNF-α per T-cell population (CD3^+^ CD4^+^ and CD3^+^ CD8^+^) after HIV stimulation (Gag, Pol/Env, Nef, and total HIV) with background subtraction (negative values obtained after removing background were imputed to zero). The percentages of cells producing at least one cytokine among IFN-γ, IL-2, and TNF-α and of polyfunctional cells (cells producing at least two or three cytokines) were also described.

### Statistical analysis.

The sample size calculation was based on the assumption that a reduction by at least 0.7 log_10_ copies/ml in plasma HIV-1 RNA level at the end of the ATI in the vaccinated group compared to the placebo group (standard deviation of the viral load at the end of the interruption estimated at 1.0 log_10_ copies/ml in the Window/ANRS 106 trial). With a two-sided type I error of 5% and a power of at least 90% (Wilcoxon rank test), the targeted numbers of participants were 35 in the placebo group and 70 in the vaccine group.

All efficacy and safety analyses were carried out as modified intention to treat (mITT), in which participants who received no vaccine dose were excluded from the analysis. The immunological analyses were based on the per-protocol population, defined as exclusion from the analyses of participants with any discontinuation of either the vaccine therapy or the ATI. Quantitative and qualitative variables were respectively described by median and interquartile range and by frequency and proportion.

The primary endpoint expressed as the maximal plasma viral load during the ATI was compared between the placebo and the vaccine groups with the two-sided Wilcoxon rank test. The immune ICS responses were compared between W0 and W28 in each arm using the Wilcoxon signed-rank test. A principal-component analysis across ICS responses at W28 (log-transformed marginal percentage of positive cells for IFN-γ, IL-2, and TNF-α per T-cell population [CD4^+^ and CD8^+^]) was performed with a projection of highest viral load during ATI, viral load slope, time to rebound, and viral load AUC as supplementary variable.

Statistical analyses were performed using SAS (version 9.3 or higher; SAS Institute, Cary, NC) and R (version 3.6.0; R Foundation for Statistical Computing, Vienna, Austria). Tests with a two-sided *P* value of <0.05 were considered statistically significant.
